# Pathology of acute sub-lethal or near-lethal irradiation of nonhuman primates prophylaxed with the nutraceutical, gamma tocotrienol

**DOI:** 10.1038/s41598-024-64102-8

**Published:** 2024-06-10

**Authors:** Vijay K. Singh, Stephen Y. Wise, Oluseyi O. Fatanmi, Sarah A. Petrus, Alana D. Carpenter, Luis A. Lugo-Roman, Sang-Ho Lee, Martin Hauer-Jensen, Thomas M. Seed

**Affiliations:** 1 Division of Radioprotectants, Department of Pharmacology and Molecular Therapeutics, F. Edward Hébert School of Medicine, Uniformed Services University of the Health Sciences, 4301 Jones Bridge Road, Bethesda, MD 20814-2712 USA; 2 Armed Forces Radiobiology Research Institute, Uniformed Services University of the Health Sciences, Bethesda, MD 20814 USA; 3 Department of Laboratory Animal Resources, Uniformed Services University of the Health Sciences, Bethesda, MD 20814 USA; 4 Pathology Department, Research Services, Naval Medical Research Center, Silver Spring, MD 20910 USA; 5 Division of Radiation Health, Department of Pharmaceutical Sciences, University of Arkansas for Medical Sciences, Little Rock, AR 72205 USA; 6 Tech Micro Services, 4417 Maple Avenue, Bethesda, MD 20814 USA

**Keywords:** Gamma-radiation, Histopathology, Linear accelerator, Nonhuman primates, Partial-body irradiation, Radiation injury, Total-body irradiation, Cell death, Drug development

## Abstract

Exposure to high, marginally lethal doses or higher of ionizing radiation, either intentional or accidental, results in injury to various organs. Currently, there is only a limited number of safe and effective radiation countermeasures approved by US Food and Drug Administration for such injuries. These approved agents are effective for only the hematopoietic component of the acute radiation syndrome and must be administered only after the exposure event: currently, there is no FDA-approved agent that can be used prophylactically. The nutraceutical, gamma-tocotrienol (GT3) has been found to be a promising radioprotector of such exposure-related injuries, especially those of a hematopoietic nature, when tested in either rodents or nonhuman primates. We investigated the nature of injuries and the possible protective effects of GT3 within select organ systems/tissues caused by both non-lethal level (4.0 Gy), as well as potentially lethal level (5.8 Gy) of ionizing radiation, delivered as total-body or partial-body exposure. Results indicated that the most severe, dose-dependent injuries occurred within those organ systems with strong self-renewing capacities (e.g., the lymphohematopoietic and gastrointestinal systems), while in other tissues (e.g., liver, kidney, lung) endowed with less self-renewal, the pathologies noted tended to be less pronounced and less dependent on the level of exposure dose or on the applied exposure regimen. The prophylactic use of the test nutraceutical, GT3, appeared to limit the extent of irradiation-associated pathology within blood forming tissues and, to some extent, within the small intestine of the gastrointestinal tract. No distinct, global pattern of bodily protection was noted with the agent’s use, although a hint of a possible radioprotective benefit was suggested not only by a lessening of apparent injury within select organ systems, but also by way of noting the lack of early onset of moribundity within select GT3-treated animals.

## Introduction

Recent global events and related military actions by select countries have resulted in ever increasing risks of radiological/nuclear devices being deployed. This dire prospect is further accentuated by increases in terrorist activity worldwide in concert with the dissemination of nuclear materials^1,2^. In sum, these situational risks serve to increase the possibility that individuals, military and civilians alike, might be exposed unwantedly to relatively high levels of ionizing radiation (IR) and, in turn, impart serious adverse health effects. The ‘radiological devices’ being referred to would certainly include tactical nuclear weapons, but also the use of radiological dispersal devices (RDD), dirty bombs or improvised nuclear devices (IND): all of these IR-emitting devices can lead to serious, life-threatening bodily injuries such as the ‘acute radiation syndrome’ (ARS)^3–6^. Such intense IR-related exposures often result in different types of multi-organ injuries requiring arrays of diagnostic tools and therapeutic interventions. Currently, there are only a limited number of agents approved by United States Food and Drug Administration (US FDA) for use as medical countermeasures (MCMs) for treatment of these unwanted exposures^7–9^. These FDA-approved MCMs include the following agents: Neupogen, Neulasta, Stimufend, Udenyca, Ziextenzo, and Leukine for treatment of acute IR-associated neutropenia and Nplate to in treat thrombocytopenia^10–20^.

Radiation MCM development following the US FDA Animal Rule requires well defined and validated animal models for radiation injury^21–25^. Various types of radiation exposures including total- and partial-body irradiation (TBI, PBI), whole thorax lung irradiation (WTLI) or abdominal irradiation have been used to induce various types and levels of IR-related injuries upon which a given medicinal can be tested for its therapeutic effect. Further, these animal models of IR injury have been used not only for MCM discovery and development, but also for the identification and validation of select surrogate biomarkers of drug safety and efficacy^26^.

In general, there are significant gaps in our knowledge relative to how these animal models respond, in turn, how the test medicinal responds, relative to the wide array of possible exposure conditions and to different types/qualities of exposing IR (e.g., gamma, X-ray, neutron, proton, or mixed field). In addition, the previously referred to ‘multi-organ’-type IR injuries requires further research in terms of the animal model being applied in MCM development.

The large nonhuman primate (NHP) animal model is a highly applicable and extraordinarily useful surrogate for studying various sub-syndromes of ARS, including the ‘delayed effects of acute radiation exposure’ (DEARE). These animals bear close resemblance to humans in terms of general anatomy and underlying commonality of basic physiology processes and genetic structure and composition (i.e., shared homology)^27–29^. It is well-known that there is > 95% DNA sequence homology between Macaques and humans^30^. Further, the NHP model of radiation injury closely resembles the clinical, and pathological response characteristics of radiation exposure-related injuries in humans. Also, similar supportive care requirements for ARS in NHPs and humans makes it easy to compare the dose effect relationships. In addition, the extended life span of NHPs is an additional attribute making this animal model quite valuable.

A member of vitamin E family, Gamma-tocotrienol (GT3, a tocol), has been extensively evaluated for its radioprotective efficacy in both murine and NHP models in several laboratories^31,32^. This agent has been selected by the Armed Forces Radiobiology Research Institute for advanced development out of eight available tocol family members as potential radiation MCM candidates^32–34^. Studies from the laboratories of several investigators have demonstrated that GT3 has significant levels of radioprotective efficacy for both hematopoietic and gastrointestinal ARS (H-ARS and GI-ARS) when investigated using murine models of ARS^31,32,35,36^. GT3 has also been shown to induce high levels of the reparative cytokine/growth factor, granulocyte colony-stimulating factor (G-GSF) and to indirectly mobilize progenitors. Furthermore, GT3 has been shown to enhance the radioprotectivness of low doses of amifostine, another important radioprotective agent to demonstrate polypharmacy approach^35,37–39^. These findings prompted us to initiate its advanced development using large animal model of NHPs^34,40–43^. Furthermore, we have also conducted studies using various omics platforms to identify its biomarkers which will be helpful to obtain regulatory approval following US FDA Animal Rule^44–51^. With additional investigation, this agent may prove to be highly useful and safe for prophylaxis against potentially ARS-related injuries. It is also important to note that within the context of the NHP model, single parenterally administered dose of GT3 without any supportive care is equivalent to multiple doses of either the already FDA-approved recombinant-based MCMs, Neupogen or to a, sustained-release formulation, Neulasta, in terms of improving hematopoietic recovery^13,17,34^. GT3 was administered once subcutaneously (*sc*) 24 h prior to irradiation with 6.5 Gy (~ LD_50/60_) or 5.8 Gy (~ LD_30/60_) gamma-radiation. In light of these observations, it is not unreasonable to suggest that GT3 might prove to be a safe and effective radioprotector for use in humans, particularly for military personnel and first responders.

In this study, we have used NHPs as a translational animal model system to investigate the histopathology of various organs from animals exposed to total- and partial-body exposure doses of 4 and 5.8 Gy. Cobalt-60 gamma-radiation and linear accelerator-derived photon radiation were used for TBI and PBI, respectively. Further, we have tested and report here the selective IR-injury mitigating effects of GT3, a component of vitamin E family and a promising MCM under advanced development. Our results demonstrate that GT3 provides varying degrees of protection to various organ systems/tissue types to two levels of radiation injury induced by either TBI or PBI.

## Materials and methods

### Experimental design and animals

In order to assess the mechanism of radioprotection by a potentially useful radiation MCM, GT3, this study was conducted using NHPs that were exposed to either a relatively low, non-lethal level (4.0 Gy), or a moderately lethal (5.8 Gy) dose. The irradiation procedure was conducted using two different sources, cobalt-60 gamma ray or a linear accelerator (LINAC)-derived photon for total- (TBI) or partial-body irradiation (PBI), respectively^44,50^.

Briefly, 31 healthy rhesus macaques (*Macaca mulatta*) 15 males and 16 females, were utilized for this study. The animals weighed from 4.0 to 8.0 kg and were 2.5–7 years of age. Randomization of the animals was accomplished by stratification using gender and body weight increments during the initial quarantine period and were then assigned to either TBI or PBI groups (16 and 15 animals assigned to the TBI and PBI groups, respectively). The grouped animals were then subsequently divided into subgroups that received either the test agent, GT3, or as a control, the agent’s vehicle alone. Out of the 16 animals within the TBI group, 8 were exposed to 4 Gy and the remaining 8 animals were exposed to 5.8 Gy. Similar grouping and subgrouping and treatments were applied to animals destined for PBI exposures. The entire study lasted for thirty days post-irradiation and all surviving animals were euthanized for tissue collection and other analysis^52,53^.

**Table:** 

4 Gy				5.8 Gy			
TBI		PBI		TBI		PBI	
Vehicle	GT3	Vehicle	GT3	Vehicle	GT3	Vehicle	GT3
Animal assignment in various treatment groups							
4	4	4	4	4	4	3	4

This animal study was conducted in a facility fully accredited by the AAALAC International. Additional details on the animals’ housing have been described earlier^34,54^. The study design and procedures received approvals from the Institutional Animal Care and Use Committee (BIOQUAL Inc. Rockville, MD and Armed Forces Radiobiology Research Institute, Uniformed Services University of the Health Sciences, Protocol #18-060) and the Department of Defense Animal Care and Use Review Office (ACURO) on 8th March 2019. The guidelines and recommendations in the *Guide for the Care and Use of Laboratory Animals* was followed strictly for conducting all procedures^55^. This study is reported in accordance with ARRIVE (Animal Research: Reporting of In Vivo Experiments) guidelines.

### Drug preparation and administration

GT3 and its vehicle were procured from Callion Pharma (Jonesborough, TN, USA). An olive oil formulation was used as the vehicle. GT3 in the vehicle was supplied as an injectable suspension at a concentration of 50 mg/ml. The quantity of GT3 and vehicle for each NHP was based on individual animal weight. Each animal received 37.5 mg/kg of GT3 or an equivalent volume of vehicle alone via *sc* injections. Details of these drug/vehicle administrations have been reported earlier^43^. In brief, drug and vehicle were administered at the dorsal scapular area (between the shoulder blades) 24 h prior to irradiation. The area surrounding the injection site was shaved at least 48 h before the administration of drug, so the site could be easily observed for any adverse skin reactions such as rash/eruption, inflammation, irritation or abscess formation following GT3 or vehicle administration. Immediately prior to drug injection, the site was wiped with 70% isopropyl rubbing alcohol and allowed to air dry; the drug was administered *sc* using a 3 ml disposable luer-lock syringe with 25-gauge 5/8-inch needle. At each injection site, injection volume was ~ 2–3 ml.

### Irradiation

Both TBI and PBI were used in this study. *TBI*: The High-Level Cobalt Facility (HLCF) used for TBI is a 35-foot square room with an internal height of 26 feet. The exposure room provides sufficient shielding for up to 400,000 curies of cobalt-60. The cobalt-60 pellets are encased in stainless steel pins that are approximately 18" long and 3/8" in diameter. The number of pins used in an irradiation depends on the required dose rate. The HLCF is categorized as a wet-storage panoramic dry irradiator, suggesting that the sources are stored underwater and raised out of the water on elevators to accomplish irradiations. There are two elevators, one on either side of a central irradiation location, allowing for both unilateral or bilateral irradiations. Typically, irradiations are performed bilateral to give the best dose uniformity through the samples. The elevators can be positioned at varying distances from the objects to achieve the required dose rate. The procedure for conducting TBI has been reported in detail previously^34^. In brief, animals were fasted for at least 12 h prior to IR exposure in order to reduce the risk of radiation-induced emesis. Two NHPs were placed on the exposure platform facing away from each other and irradiated with midline doses of either 4.0 or 5.8 Gy at a dose rate of 0.6 Gy/min from both sides of the animal’s abdominal core (bilateral, simultaneous exposure). The abdominal width of each animal was measured with digital calipers. The radiation dosimetry details have been provided earlier^54^. During the exposure, the mildly sedated and conscious animals were observed via in-room monitors. After the procedure, animals were maintained in a staging area, until they were sufficiently alert to allow for a return to their cage, where full recovery was completed. *PBI*: Similar to TBI, the PBI procedure has been described earlier^40^. In brief, food was withheld from the animal for about 12 h before the procedure. In contrast to TBI, the animals receiving PBI were fully sedated, as the use of a restraining device was not possible. Animals were singularly irradiated at the dose-rate of 1.3 Gy/min using a 4 MV photon beam from an Elekta Infinity clinical LINAC. Prior to the exposures, both the IR dose and animal’s ID were verified by the attending dosimetrist of the LINAC facility. Select vital signs (e.g., heart rates and blood oxygen levels) of the irradiated animals were monitored using SurgiVet Advisor tech Vital Signs Monitor (Smiths Medical, Dublin, OH, USA). In order to achieve a ~ 5% sparing of bone marrow, the hind limbs, fibula, tibia, and feet were excluded from the radiation field. To accurately deliver the dose to the animal at the location of the desired target (abdominal core), the anterior/posterior (AP) measurements of the animal’s abdomen were accomplished with digital calipers. Near the field edge at which the knees were positioned, approximately 1 cm of the spared limb was in the penumbra region (80% to 20% dose of the field center). The rest of the spared limb received absorbed doses below 20% of the field center.

### Supportive care

After any procedures, NHPs were monitored at least twice daily for signs of complications. The type of supportive care provided was based on CBC and cage side observations^56^. The primary antibiotic used was enrofloxacin (Baytril Bayer HealthCare LLC, Shawnee Mission, KS). The dose administered was 5 + 0.25 mg/kg intramuscularly (*im*) or *sc* twice a day (BID); or 10 + 0.5 mg/kg administered *im* or intravenous (*iv*) once daily (QD). Additional supportive care measures included rehydration fluids, alternate antibiotics, antipyretics, antidiarrheal agents, analgesics, antiemetics, and nutritional support. Details of supportive care is described in detail earlier^53^.

### Clinical assessments

General behavioral observations were recorded daily for each animal under test. This included noting any/all changes in urination/defecation patterns, along with periodic measures of body weights, heart rates, and blood pressures. Conventional hematological assessments were also conduced. Details of the blood drawing procedures and related CBC analyses were previously reported^43^. Standard CBC analyses were performed using a Bayer Advia 120 cell counter (Siemens, Malvern, PA, USA).

### Euthanasia

Moribundity was used as a surrogate endpoint for mortality. In select cases, euthanasia was carried out prior to the study’s designated duration of 30 days post-exposure in order to reduce undue pain and suffering for animals (2 animals at 21 & 22 d following 5.8 Gy radiation exposures were found to be moribund). When an animal reached a state of moribundity, the animal was immediately euthanized. Euthanasia was conducted in line with the directives of the approved IACUC protocol, the Guide for the Care and Use of Laboratory Animals (8th edition), and the American Veterinary Medical Association (AVMA) Guidelines for the Euthanasia of Animals (2020 Edition), with the details of the parameters and guidelines previously described^55,56^. The moribund animals were given pentobarbital sodium *iv* (Virbac AH Inc., Fort Worth, TX) using either saphenous or cephalic veins, needle size 20–25 gauge (100 mg/kg, 1–5 ml). Prior to pentobarbital sodium administration, animals were sedated using ketamine hydrochloride injection (Mylan Institutional LLC, Rockford, IL) (5–15 mg/kg, *im*). Intra-cardiac administration was performed if unable to administer pentobarbital sodium through peripheral veins. The animals were deeply anesthetized by Isoflurane (Baxter Healthcare Corporation, Deerfield, IL) (1–5%) with oxygen at 1–4 L per minute via mask before administering the intra-cardiac injection. The animals were euthanized only under the guidance of a staff veterinarian or a trained technician in consultation with the veterinarian. After pentobarbital sodium administration, the animals were examined by assessing the heart auscultation and pulse to confirm death.

### Necropsy

Following euthanasia, tissue samples were harvested under the direction of a board-certified veterinary pathologist and all observed gross abnormalities were recorded during the procedure. The harvested tissues, including sternum, spleen, duodenum, jejunum, ileum, colon, liver, kidney, and lung, were loaded into histology cassettes and subsequently placed in containers and fixed in 10% buffered zinc formalin. Collected and fixed tissues were embedded, processed and sectioned using standard histological procedures as described earlier^53^.

### Histopathology

Zinc-buffered formalin fixed NHP tissues were subsequently processed for paraffin embedding, sectioning, and hematoxylin and eosin (H&E) staining of prepared slides (via Histoserv, Inc, Germantown, MD, USA)^57^. The histological scoring and morphometric analyses were conducted by a board-certified veterinary pathologist. The scoring system, i.e., the grading system, employed was based on a point system of noted pathology within the observed tissue; namely, 0 = none, 1 = minimal, 2 = mild, 3 = moderate, 4 = marked, and 5 = severe. Digital images of tissue sections were obtained with a Ziess Axioscan slide scanner and Zeiss Zen software (Carl Ziess Meditech, Inc., Dublin, CA, USA). An Olympus IX73 microscope (Olympus, Center Valley, PA) was used for 40× magnification. Histological and morphometric analyses were conducted under blinded conditions related to given treatment groups.

### Statistical analysis

Statistical analysis of CBC and vital signs data was accomplished using statistical software IBM SPSS Statistics (version 28.0.1.1, https://www.ibm.com/products/spss-statistics). All error bars signify standard deviations. For CBC data, One-Way ANOVA tests were performed between GT3-treated groups and their respective vehicle groups (i.e., PBI GT3 vs. PBI Vehicle, and TBI GT3 vs. TBI Vehicle), vehicle-treated groups (i.e., PBI Vehicle vs. TBI Vehicle), and GT3-treated groups (i.e., PBI GT3 vs. TBI GT3). *P*-values < 0.05 were considered statistically significant for all tests performed.

### Ethics statement

All procedures were approved by the Institutional Animal Care and Use Committee Armed Forces Radiobiology Research Institute/Uniformed Services University of the Health Sciences and BIOQUAL Inc., Rockville, and Department of Defense Animal Care and Use Review Office (ACURO). This study was carried out in strict accordance with the *Guide for the Care and Use of Laboratory Animals* of the National Institutes of Health.

## Results

### Clinical assessments

*General observations.* General clinical/behavioral/pathological observations were made on all test animals throughout the study period (i.e., − 8 days to 30 days). With the exception of the two ‘vehicle control’ animals receiving 5.8 Gy TBI and that became moribund prior to the scheduled end of the test period, the most frequent behavioral change that manifest acute radiation illness was a ‘back-hunching’ response seen at the higher, sublethal dose tested (5.8 Gy). Primary metrics of clinical status are described with major findings listed. In brief, body weight (Table 1), body temperature (Table 2), heart rate (Table 3), blood pressure (Table 4), gross pathology (Table 5), and histopathology (Table 6) of all irradiated animals are listed. In aggregate, these data indicate varying, but mostly modest degrees of change in clinical status following IR-exposure. By contrast however, the clinical condition of several animals changed rather markedly during the initial post-exposure period.

**Table 1:** Body weights of NHPs subjected to 4 or 5.8 Gy TBI or PBI and treated with GT3 or vehicle.

Radiation dose	Exposure level	Treatment	Age (years)	(N size), Average % Change ± SD		
				Total	Males	Females
4 Gy	PBI	Vehicle	3.6 ± 0.5	(4) − 0.20 ± 1.86	(2) − 0.09 ± 1.44	(2) − 0.31 ± 2.87
		GT3	3.8 ± 0.1	(4) 2.22 ± 6.00	(2) − 0.58 ± 1.86	(2) 5.02 ± 8.54
	TBI	Vehicle	4.0 ± 0.6	(4) − 2.59 ± 2.59	(2) − 2.52 ± 2.43	(2) − 2.66 ± 3.77
		GT3	4.0 ± 0.7	(4) 0.48 ± 5.73	(2) 4.75 ± 1.64	(2) 3.79 ± 4.78
5.8 Gy	PBI	Vehicle	3.6 ± 0.2	(3) 0.30 ± 3.08	(1) 1.11	(2) − 0.10 ± 4.24
		GT3	3.4 ± 0.6	(4) 1.04 ± 7.00	(2) − 2.79 ± 2.84	(2) 4.87 ± 8.95
	TBI	Vehicle	3.6 ± 0.2	(4) − 5.25 ± 7.46	(2) − 3.57 ± 3.34	(2) − 6.93 ± 12.01
		GT3	3.6 ± 0.5	(4) − 1.93 ± 2.84	(2) − 2.36 ± 4.59	(2) − 1.51 ± 1.54

± , standard deviation.

**Table 2:** Temperatures of NHPs subjected to 4 or 5.8 Gy TBI or PBI and treated with GT3 or vehicle.

Radiation dose	Exposure level	Treatment	Average temperature (Celsius) ± SD recorded post-irradiation (d)											
			− 8	2	7	14	16	18	20	22	24	26	28	30
4 Gy	PBI	Vehicle	38.38 ± 0.60	38.11 ± 0.67	38.82 ± 0.50	38.63 ± 0.51	39.00 ± 0.32	38.99 ± 0.66	39.04 ± 0.53	39.29 ± 0.52	39.24 ± 0.41	39.41 ± 0.74	38.96 ± 0.37	39.04 ± 0.90
		GT3	38.79 ± 0.23	38.88 ± 0.27	39.49 ± 0.41	39.38 ± 0.29	39.39 ± 0.72	39.60 ± 0.39	39.33 ± 0.56	39.56 ± 0.45	39.39 ± 0.69	39.07 ± 1.02	39.11 ± 1.00	39.79 ± 0.64
	TBI	Vehicle	38.96 ± 1.19	39.13 ± 0.60	38.54 ± 0.73	38.85 ± 0.45	38.47 ± 0.84	38.28 ± 1.18	39.11 ± 0.16	38.95 ± 0.33	38.48 ± 1.01	38.88 ± 0.67	39.15 ± 0.90	38.82 ± 0.61
		GT3	39.14 ± 0.52	38.88 ± 0.31	38.95 ± 0.44	38.85 ± 0.09	38.69 ± 0.81	38.64 ± 0.88	39.18 ± 0.34	39.18 ± 0.67	38.81 ± 0.91	39.01 ± 0.62	38.91 ± 0.88	39.17 ± 0.15
5.8 Gy	PBI	Vehicle	38.78 ± 0.31	37.08 ± 1.15	37.02 ± 0.12	37.44 ± 0.43	39.48 ± 0.51	39.43 ± 0.32	39.26 ± 0.57	39.45 ± 0.48	40.13 ± 0.54	40.74 ± 0.19	40.04 ± 0.75	38.77 ± 0.54
		GT3	39.03 ± 0.24	37.69 ± 1.02	38.52 ± 0.37	37.63 ± 0.63	39.19 ± 0.79	39.35 ± 0.56	39.31 ± 0.39	39.49 ± 0.58	39.92 ± 0.65	40.15 ± 0.62	40.12 ± 0.67	38.71 ± 0.66
	TBI	Vehicle	37.96 ± 0.52	38.67 ± 0.32	38.45 ± 0.55	38.42 ± 0.72	39.47 ± 0.44	39.74 ± 0.43	39.56 ± 0.87	40.28 ± 0.31	40.36 ± 0.59	40.73 ± 1.18	39.92 ± 1.61	38.06 ± 0.47
		GT3	38.85 ± 0.89	37.79 ± 0.64	38.64 ± 0.34	38.39 ± 0.89	39.47 ± 0.41	39.49 ± 0.92	39.37 ± 0.42	39.79 ± 0.41	40.13 ± 0.70	40.03 ± 1.15	40.00 ± 0.70	38.71 ± 0.60

d, day; ± , standard deviation.

**Table 3:** Heart rates of NHPs subjected to 4 or 5.8 Gy TBI or PBI and treated with GT3 or vehicle.

Radiation Dose	Exposure Level	Treatment	Average Heart Rate (BPM) recorded post-irradiation (d)							
			− 8	16	18	20	22	24	26	28
4 Gy	PBI	Vehicle	249 ± 13	221 ± 25	236 ± 25	222 ± 53	189 ± 34	188 ± 43	213 ± 46	190 ± 36
		GT3	225 ± 31	207 ± 17	228 ± 23	214 ± 22	196 ± 44	176 ± 49	224 ± 69	190 ± 34
	TBI	Vehicle	ND	180 ± 97	193 ± 0	161 ± 59	217 ± 40	187 ± 71	219 ± 35	195 ± 57
		GT3	ND	254 ± 24	236 ± 12	205 ± 41	229 ± 30	235 ± 23	224 ± 30	211 ± 11
5.8 Gy	PBI	Vehicle	245 ± 16	203 ± 8	238 ± 19	220 ± 14	197 ± 2	217 ± 26	205 ± 5	209 ± 12
		GT3	224 ± 12	225 ± 20	235 ± 10	203 ± 12	209 ± 9	206 ± 15	217 ± 18	210 ± 15
	TBI	Vehicle	ND	234 ± 35	232 ± 50	224 ± 27	162 ± 45	197 ± 11	194 ± 57	213 ± 101
		GT3	ND	221 ± 19	234 ± 31	171 ± 50	204 ± 39	214 ± 33	209 ± 22	181 ± 5

d, day; ND, no data; ± , standard deviation.

**Table 4:** Blood pressures of NHPs subjected to 4 or 5.8 Gy TBI or PBI and treated with GT3 or vehicle.

Radiation dose	Exposure level	Treatment	Average blood pressure recorded post-irradiation (d)							
			− 8	16	18	20	22	24	26	28
4 Gy	PBI	Vehicle	143/87 ± 26/27	132/92 ± 20/11	149/82 ± 22/24	142/91 ± 19/8	145/98 ± 21/18	153/108 ± 20/11	152/89 ± 30/18	125/89 ± 12/12
		GT3	148/94 ± 28/21	133/78 ± 24/35	135/77 ± 28/13	133/85 ± 18/9	129/84 ± 34/29	140/80 ± 16/17	150/80 ± 24/5	132/82 ± 5/13
	TBI	Vehicle	ND	134/90 ± 7/11	99/70 ± 16/13	111/80 ± 62/49	116/73 ± 42/46	162/110 ± 11/6	122/55 ± 30/11	131/59 ± 13/13
		GT3	ND	114/80 ± 35/28	118/84 ± 18/5	125/88 ± 11/5	124/72 ± 32/1	145/78 ± 28/11	129/85 ± 11/14	121/88 ± 7/1
5.8 Gy	PBI	Vehicle	133/86 ± 8/7	140/80 ± 9/14	151/91 ± 21/30	134/79 ± 23/21	127/88 ± 11/9	126/78 ± 12/4	119/74 ± 7/4	126/67 ± 10/1
		GT3	134/69 ± 19/8	129/78 ± 4/16	136/82 ± 11/17	128/92 ± 18/23	130/86 ± 12/6	137/97 ± 27/27	129/68 ± 17/18	135/71 ± 27/18
	TBI	Vehicle	ND	107/78 ± 23/20	98/53 ± 32/15	84/49 ± 10/13	116/91 ± 31/41	124/61 ± 11/18	113/82 ± 6/7	106/65 ± 1/5
		GT3	ND	114/72 ± 19/14	95/66 ± 35/34	133/85 ± 5/17	127/96 ± 6/8	120/77 ± 20/20	104/62 ± 33/13	125/80 ± 25/30

d, day; ND, no data; ± , standard deviation.

**Table 5:** Gross pathological findings of NHP tissues from animals exposed to 4 or 5.8 Gy TBI or PBI and treated with GT3 or vehicle.

Radiation dose	Exposure level	Treatment	Euthanasia (d)	NHP#	Sex	Age (years)	Gross pathological findings
4 Gy	PBI	Vehicle	30	1603102	F	3.9	Kidneys appear pale, mild hemorrhage on both lungs. No other gross abnormalities were noted. BCS 2.5/5 (thin)
				1707119	M	3.8	Minute petechiae noted in GI tract, some hemorrhage noted in both lungs. BCS 3/5 (ideal)
				1704305	M	2.8	Mild to moderate hemorrhage noted in abdominal cavity. BCS 3/5 (ideal)
				1603026	F	3.9	Approximately 5% dehydrated, shriveled spleen, pale kidneys. No other gross abnormalities noted. BCS 2.5/5 (lean)
		GT3		1603079	M	3.9	Overall, animal was unremarkable. Few petechiae noted in few locations along the GI tract. BCS 3/5 (ideal)
				1704119	M	3.8	In general, animal was unremarkable. BCS 3/5) ideal
				1606088	F	3.6	Petechiae noted on the distal portion of bladder. BCS 3/5 (ideal)
				1603168	F	3.9	Animal appears dehydrated, mild hemorrhage noted on the ileo-cecal junction. BCS 1.5/5 (thin)
	TBI	Vehicle	30	RA2695	M	4.7	Overall, animal appeared unremarkable, few to moderate petechiae noted in the stomach and duodenum. BCS 3/5 (ideal)
				1606069	M	3.6	Moderate pallor at the lung margins, few petechiae noted on both lobes with moderate focal hemorrhage and blotched serrations across the surface of the spleen accompanied with ulcerative-like lesions. Splenic exhaustion noted from gross presentation, approximately 7% dehydrated. BCS 3/5 (ideal)
				RA3228	F	4.1	Apparent dehydrations presented with pale kidneys, shriveled spleen, petechiae/hemorrhage in jejunum and ileum, and bloated colon. BCS 2.5/5 (lean)
				1608010	F	3.5	Severe, diffuse, hemorrhagic pneumonitis with adhesions noted in lungs. Moderate cardiomegaly noted with significant hemorrhage on the proximal section of the heart. Less than 5% dehydrated, pale mucous membranes, and intussusceptions along the GI tract. BCS 2.5/5 (lean)
		GT3		RA3275	M	3.9	Slight dehydration presented with pale spleen, kidneys, and liver. Moderate petechiae noted in small and large intestines. BCS 2.5/5 (lean)
				1606093	M	3.6	Moderate pallor at the lung margins with few hemorrhages noted on the distal lobes of both lungs and moderate focal hemorrhage noted on and across the surface of the spleen accompanied with ulcerative-like lesions. Splenic exhaustion noted from gross with shriveled appearance. Approximately 7% dehydrated with pale mucous membranes. BCS 3/5 (ideal)
				RA2770	F	5.1	Mild to moderate swelling of right antebrachium region and hand of unknown origin. No other gross abnormalities were noted. BCS 2.5/5 (lean)
				1607012	F	3.5	Mild pallor at the margins of the lungs. Approximately 5–7% dehydrated with pale mucous membranes. BCS 3/5 (ideal)
5.8 Gy	PBI	Vehicle	30	1608017	M	3.5	Cardiac lesion of unknown cause presented with hemorrhages and petechiae noted on the surface and inside if the heart, enlarged mediastinal lymph nodes with several fat deposits. Moderately distended urinary bladder with few petechiae noted on its surface. BCS 2.5/5 (slightly thin)
				1608018	F	3.5	Interventricular septum hypertrophy, swelling on the right side of the heart with moderate petechiae noted on the surface of the heart, pale kidneys, intussusceptions in the GI tract. No other remarkable gross findings. BCS 2/5 (thin)
				1604188	F	3.8	Moderate dehydration presented with mild pale margins of the lung lobes, few adhesions noted on the upper and middle lobes of right lung, petechiae on both lungs. Mild hemorrhage throughout the small intestines with few intussusceptions. Approximately 7% dehydrated with pale mucus membranes and pale kidneys. BCS 2.5/5 (lean)
		GT3		1608007	M	3.5	Roughened, mottled edges of the spleen with shriveled appearance, less than 5% dehydrated with pale mucus membranes. BCS 2.5/5 (lean)
				1708011	M	2.5	Moderate generalized hemorrhagic enteritis, few petechiae/hemorrhages noted in small and large intestines no other remarkable gross findings. 5–7% dehydrated with pale mucus membranes. BCS 2/5 (thin)
				1603104	F	3.9	Enlarged right ventricle with roughened heart surface, swollen mediastinal lymph nodes. No other gross abnormalities noted. BCS 2/5 (thin)
				1608006	F	3.5	Enlarged spleen with no other remarkable gross findings. 5–7% dehydrated with pale mucus membranes. BCS 3/5
	TBI	Vehicle	30	1608039	M	3.5	Moderate focal hemorrhagic lesions towards margins of the lungs, some adhesions, petechiae, and hemorrhages noted in both lungs. Globoid heart with increased pericardial effusion, roughened heart surface with moderate hemorrhages noted on the surface of the heart. Moderate focal hemorrhage of the left renal cortex and on parietal surface beneath the capsule, both kidneys appear pale. BCS 3/5 (lean)
				1603015	M	3.9	Pallor noted at the margins of the lung lobes, mild pallor of the liver, inflamed gall bladder with greenish grainy bile fluid. Some petechiae and hemorrhages noted in small and large intestines No other remarkable gross findings. Approximately 7% dehydrated. BCS 3/5 (lean)
			22	1607030	F	3.5	Marked pallor of mucus membranes suggestive of dehydration, generalized pallor noted throughout lungs with multifocal hemorrhagic necrosis and moderate petechiae noted in both lungs. Severe pericardial edema and pallor of the heart tissue, with some serrations noted on the cardiac surface. Enlarged mesenteric lymph nodes with petechiae in small and large intestines, and enlarged left kidney with focal area of hemorrhagic necrosis at the cortex. Both kidneys appear pale. Severe hemorrhagic lesion and intussusception at the ileo-cecal junction, several petechiae along the jejunal surface. Approximately 10–12% dehydrated. BCS 1.5/5 (very thin)
			21	1608016	F	3.5	Generalized pallor throughout lungs, with both lungs presented with petechiae, hemorrhages, and some adhesions in few of the lobes. Serrated surface of the heart presented with moderate hemorrhagic carditis at the left ventricle, moderate pericardial edema, multifocal petechial hemorrhage on the surface of the heart, and some blood clots on the inside of the heart. Moderate pallor of the left kidney with both kidneys looking pale, multifocal necrotic lesions of the spleen with a shriveled appearance. GI Tract presented with lesions, hemorrhages, and petechiae, with intussusceptions noted in the small intestines. Less than 5% dehydrated with marked pallor of mucous membranes. BCS 2.5/5 (thin)
		GT3	30	1703269	M	2.9	No remarkable gross findings. Mild to moderate petechiae noted in GI tract. Approximately 7% dehydrated with pale mucous membranes. BCS 3/5 (normal)
				1607029	M	3.5	Diffuse hemorrhagic congestion of the lungs, more prevalent throughout left lung field, with both lungs presented with some petechiae and adhesions. Stomach slightly distended with some bloating noted in the large intestine. Approximately 7% dehydrated with pale mucous membranes. BCS 2.5/5 (lean)
				1603138	F	3.9	Diffuse petechial hemorrhage of the lungs with pale margins of the lobes, with mild to moderate petechiae in both lungs. Golf ball sized ovarian mass discovered at necropsy, no history of clinical signs relative to this finding. Moderate petechiae noted in bladder. Approximately 7% dehydrated with pale mucous membranes. BCS 3/5 (ideal)
				1603030	F	3.9	Mild pale margins of the lung lobes, mild hemorrhage throughout small intestine with few intussusceptions noted in small and large intestines. Approximately 7% dehydrated with pale mucous membranes. BCS 2.5/5 (lean)

The body condition score (BCS) is a subjective method of gaging body fat and muscle by palpation. All scores were provided by the veterinarian at the time of necropsy. The scale used both whole and half units, an optimal body condition would be scored a 3.0. Lower scores represent emaciated to lean conditions (1.0 to 2.0), and higher values (4.0 to 5.0) indicate excessive body fat.

**Table 6:** Histopathological scoring of various organs of healthy animals in addition to TBI and PBI animals treated with either GT3 or vehicle.

Organs	Average histopathology scores								
	Vehicle				GT3				Healthy (n = 3)
	4 Gy		5.8 Gy		4 Gy		5.8 Gy		
	TBI (n = 4)	PBI (n = 4)	TBI (n = 4)	PBI (n = 3)	TBI (n = 4)	PBI (n = 4)	TBI (n = 4)	PBI (n = 4)	
Lung									
Alveolar septal degeneration	2 ± 1	1 ± 0	2 ± 1	1 ± 1	1 ± 1	2 ± 1	1 ± 1	2 ± 1	1 ± 1
Alveolar edema	0 ± 1	0 ± 0	1 ± 1	0 ± 0	0 ± 0	0 ± 0	1 ± 1	0 ± 0	1 ± 2
Septal cellularity	0 ± 0	0 ± 0	1 ± 2	0 ± 0	0 ± 0	0 ± 0	0 ± 0	0 ± 0	0 ± 0
Kidney									
Tubular degeneration	3 ± 1	2 ± 0	3 ± 0	3 ± 0	2 ± 1	2 ± 1	3 ± 1	3 ± 1	3 ± 1
Tubular regeneration	0 ± 0	0 ± 0	0 ± 1	0 ± 0	0 ± 0	0 ± 1	0 ± 0	0 ± 0	1 ± 1
Cellular infiltrates	0 ± 0	0 ± 1	0 ± 0	1 ± 1	0 ± 1	0 ± 1	1 ± 1	1 ± 1	0 ± 0
Sternum									
Cellular depletion	0 ± 1	1 ± 1	2 ± 2	1 ± 1	1 ± 1	3 ± 1	1 ± 1	2 ± 1	1 ± 1
Spleen									
White pulp depletion	3 ± 1	2 ± 1	4 ± 0	3 ± 1	3 ± 2	2 ± 1	3 ± 0	2 ± 1	1 ± 1
Duodenum									
Villi/crypt ratio	4	3 ± 1	2 ± 0	3 ± 1	4	3 ± 1	3 ± 1	3 ± 0	4 ± 1
Inflammatory cell infiltrates	2 ± 1	1 ± 1	1 ± 1	2 ± 1	2 ± 1	1 ± 1	2 ± 1	2 ± 1	0 ± 0
Crypt dilation	1 ± 1	0 ± 0	0 ± 1	0 ± 0	0 ± 0	0 ± 0	1 ± 1	0 ± 0	0 ± 0
Villi fusion	2 ± 1	2 ± 1	2 ± 1	1 ± 0	1 ± 1	1 ± 0	1 ± 1	1 ± 1	1 ± 1
Villi loss	3 ± 0	2 ± 1	3 ± 1	2 ± 1	3 ± 1	3 ± 1	2 ± 1	2 ± 1	0 ± 0
Jejunum									
Villi/crypt ratio	4 ± 2	5 ± 1	4 ± 1	4 ± 2	4 ± 1	4 ± 1	4 ± 1	4 ± 1	4 ± 1
Inflammatory cell infiltrates	0 ± 1	1 ± 1	0 ± 0	1 ± 1	1 ± 1	1 ± 1	1 ± 1	1 ± 1	1 ± 1
Crypt dilation	0 ± 0	0 ± 0	1 ± 1	0 ± 1	0 ± 0	0 ± 0	0 ± 0	0 ± 0	0 ± 0
Villi fusion	1 ± 1	1 ± 1	2 ± 0	1 ± 1	1 ± 1	1 ± 1	1 ± 1	1 ± 1	1 ± 0
Villi loss	2 ± 1	2 ± 1	3 ± 1	2 ± 1	2 ± 1	1 ± 1	2 ± 0	3 ± 1	1 ± 1
Ileum									
Villi/crypt ratio	5 ± 1	4 ± 1	4 ± 1	5 ± 1	4 ± 1	4 ± 0	4 ± 1	4 ± 1	5
Inflammatory cell infiltrates	0 ± 1	1 ± 1	0 ± 1	1 ± 1	0 ± 0	1 ± 1	1 ± 1	0 ± 1	0 ± 0
Crypt dilation	0 ± 0	0 ± 0	0 ± 1	0 ± 0	0 ± 0	0 ± 0	0 ± 0	0 ± 0	0 ± 0
Villi fusion	2 ± 1	2 ± 0	2 ± 1	1 ± 1	2 ± 1	2 ± 1	1 ± 1	2 ± 1	1 ± 1
Villi loss	3 ± 1	2 ± 1	3 ± 1	3 ± 0	3 ± 1	3 ± 1	3 ± 1	2 ± 1	2 ± 1
Large intestine									
Inflammatory cell infiltrates	2 ± 1	1 ± 0	2 ± 1	2 ± 0	1 ± 1	2 ± 1	2 ± 1	2 ± 1	1 ± 0
Crypt dilation	0 ± 0	0 ± 0	0 ± 0	0 ± 1	0 ± 0	0 ± 0	0 ± 0	0 ± 0	0 ± 0
Crypt loss	3 ± 1	3 ± 1	2 ± 1	2 ± 1	2 ± 1	2 ± 2	2 ± 1	2 ± 1	3 ± 1
Liver									
Inflammatory cell infiltrates	0 ± 1	0 ± 0	1 ± 1	0 ± 0	0 ± 1	0 ± 0	0 ± 1	0 ± 1	0 ± 0
Cystic degeneration	1 ± 1	2 ± 1	2 ± 1	2 ± 1	1 ± 1	2 ± 1	2 ± 1	2 ± 1	1 ± 0

± , standard deviation; PBI, partial-body irradiation; TBI, total-body irradiation.

*Body weight.* Significant shifts in body weights (± 10%) were not noted generally in animals subjected to the different IR exposure levels (4 or 5.8 Gy), or to the two different exposure protocols (TBI or PBI), or the types of treatments (vehicle or GT3). The two exceptions to the later were noted however and both cases were observed at the higher level of exposure (5.8 Gy): the first animal, pretreated with GT3 and subjected to PBI, exhibited a weight gain of ~ 11% at 30 d post-exposure (i.e., at the time of euthanasia), while the second animal, pretreated with the control ‘vehicle’ alone and exposed TBI, showed significant weight loss (~ 15%) at 21 d post exposure. This animal was deemed moribund and in turn euthanized. The average body weight of all animals under test prior to IR exposure was 5.24 kg (SD = 0.994), whereas the average weight at the time of euthanasia was 5.252 (SD = 0.934) (Table 1, Supplementary Table 1). Body weights of the GT3 and vehicle-treated animals over the test period did not demonstrate significant difference.

*Temperature.* No significant shifts in body temperatures were recorded for the entire cohort or various subsets test animals subjected to the various doses and regimens of IR exposure or to the two different pretreatments. The average body temperature of all animals under test prior to IR exposure was 38.7 C (SD = 0.68), whereas the average body temperature at the time of euthanasia was 39.0 C (SD = 0.68) (Table 2, Supplementary Table 2).

*Heart rate and blood pressure.* ‘Heart rate’ along with ‘Blood pressure’ measurements were monitored throughout the entire test period. For both these clinical assessments, no clear patterns were noted relative to the type of IR exposure, level of those exposures, or the type of medicinal being tested. The average heart rate of 15 (out of 31) animals under test prior to IR exposure was 235 beats/min (SD = 0.21), whereas the average heart rate at the time of euthanasia was 198 beats/min (SD = 0.33) for 25 of the 31 animals under test (Table 3, Supplementary Table 3). The average blood pressure measurements (systolic/diastolic readings) of animals under test under test prior to IR exposure (for 15/31 animals monitored) were 140/84 (SDs = 21/19), whereas those average readings at the time of euthanasia were 126/76 (SD2 = 16/17) (for 25/31 animals) (Table 4, Supplementary Table 4).

**Figure 1:**
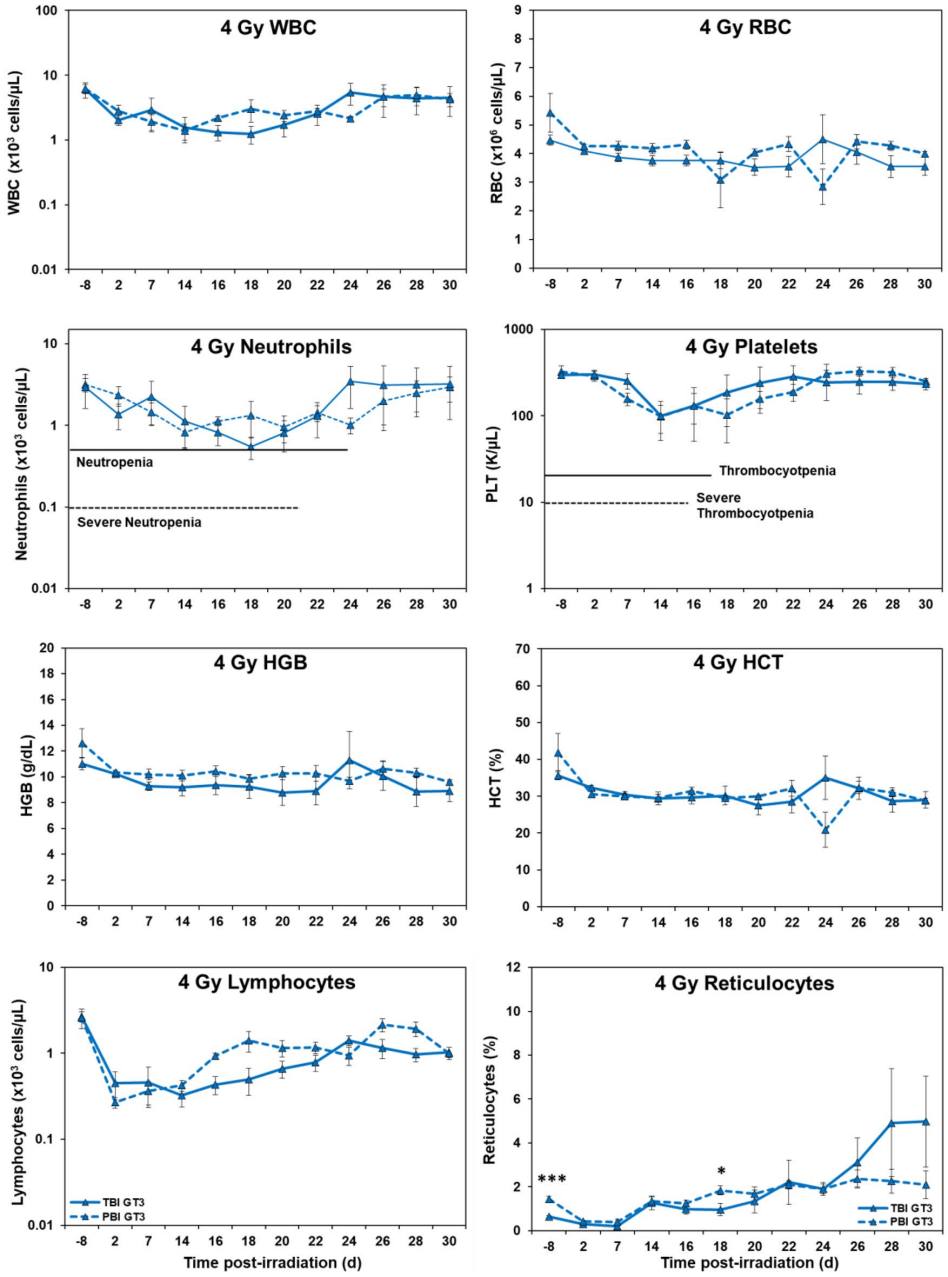
Effects of 4 Gy total-body and partial-body irradiation on GT3-treated NHPs. The data for each time point is presented as the mean for each group. There was a total of 8 NHPs; 4 in the TBI group and 4 in the PBI group. Analysis of variance was performed with SPSS to detect significant differences between TBI and PBI groups. The asterisk corresponds to a statistical significance of *p* < 0.05.

**Figure 2:**
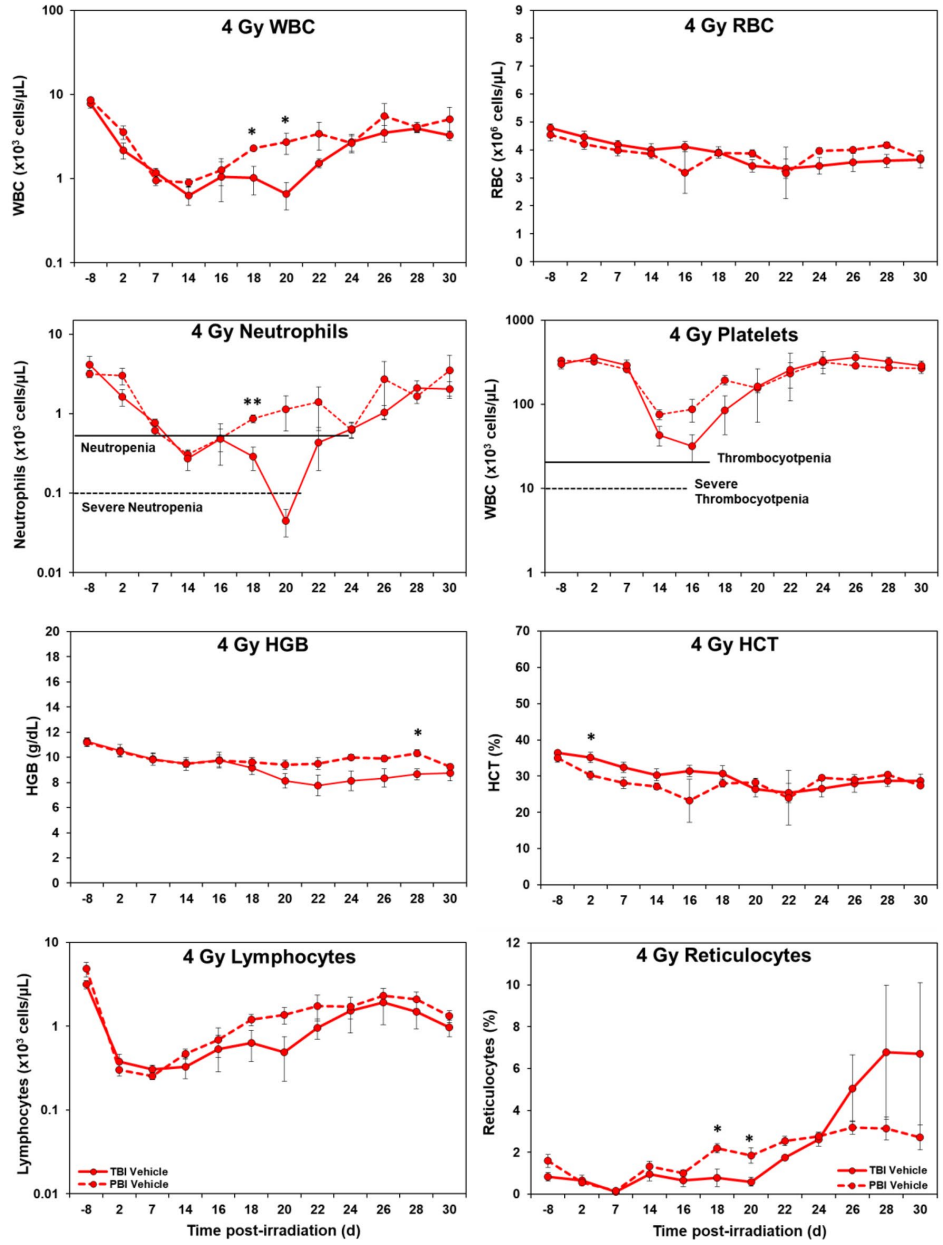
Effects of 4 Gy total-body and partial-body irradiation on vehicle-treated NHPs. The data for each time point is presented as the mean for each group. There was a total of 8 NHPs; 4 in the TBI group and 4 in the PBI group. Analysis of variance was performed with SPSS to detect significant differences between TBI and PBI groups. The number of asterisks corresponds to the level of statistical significance as follows: *p < 0.05, **p < 0.01.

**Figure 3:**
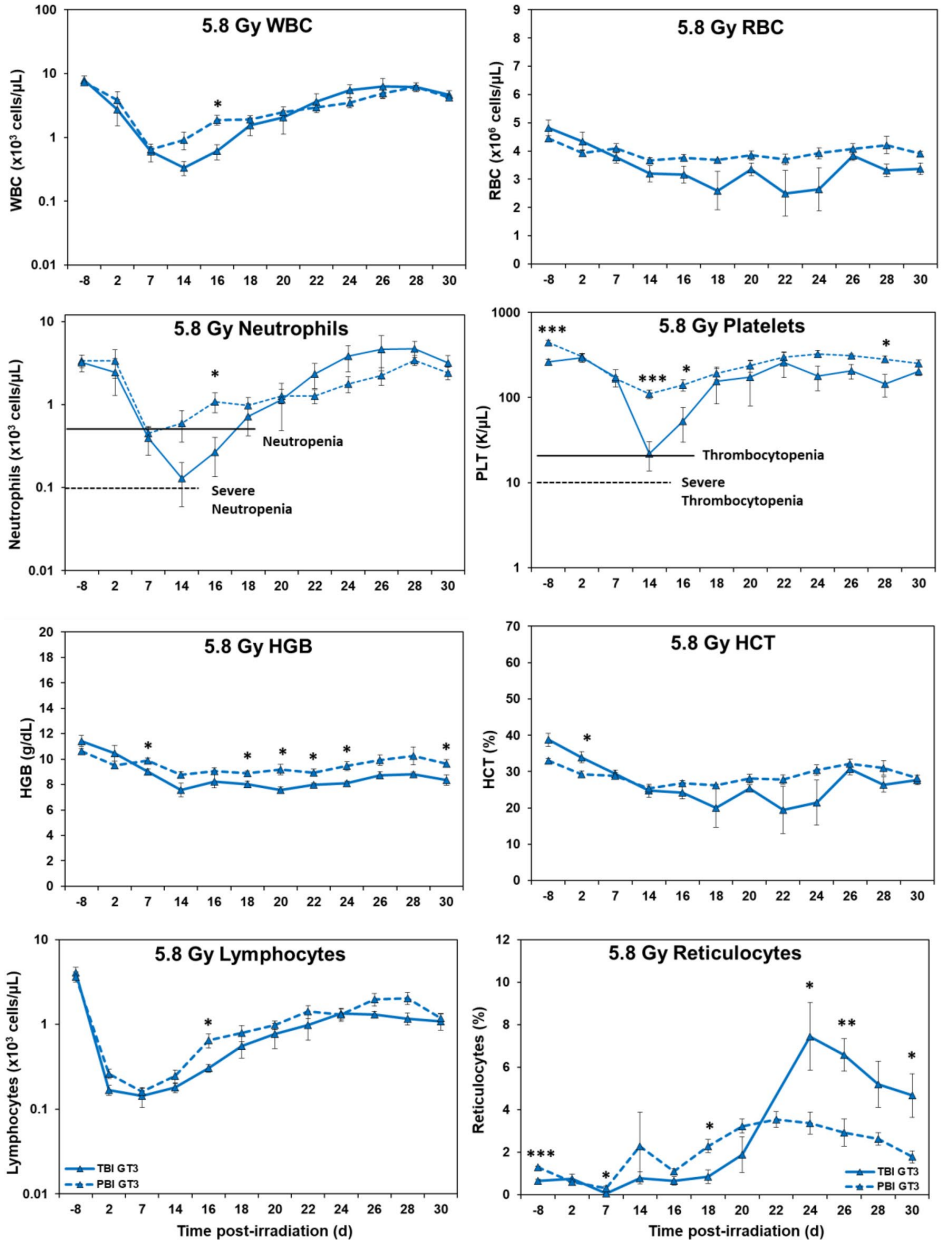
Effects of 5.8 Gy total-body and partial-body irradiation on GT3-treated NHPs. The data for each time point is presented as the mean for each group. There was a total of 8 NHPs; 4 in the TBI group and 4 in the PBI group. Analysis of variance was performed with SPSS to detect significant differences between TBI and PBI groups. The number of asterisks corresponds to the level of statistical significance as follows: *p < 0.05, **p < 0.01, ***p < 0.001.

**Figure 4:**
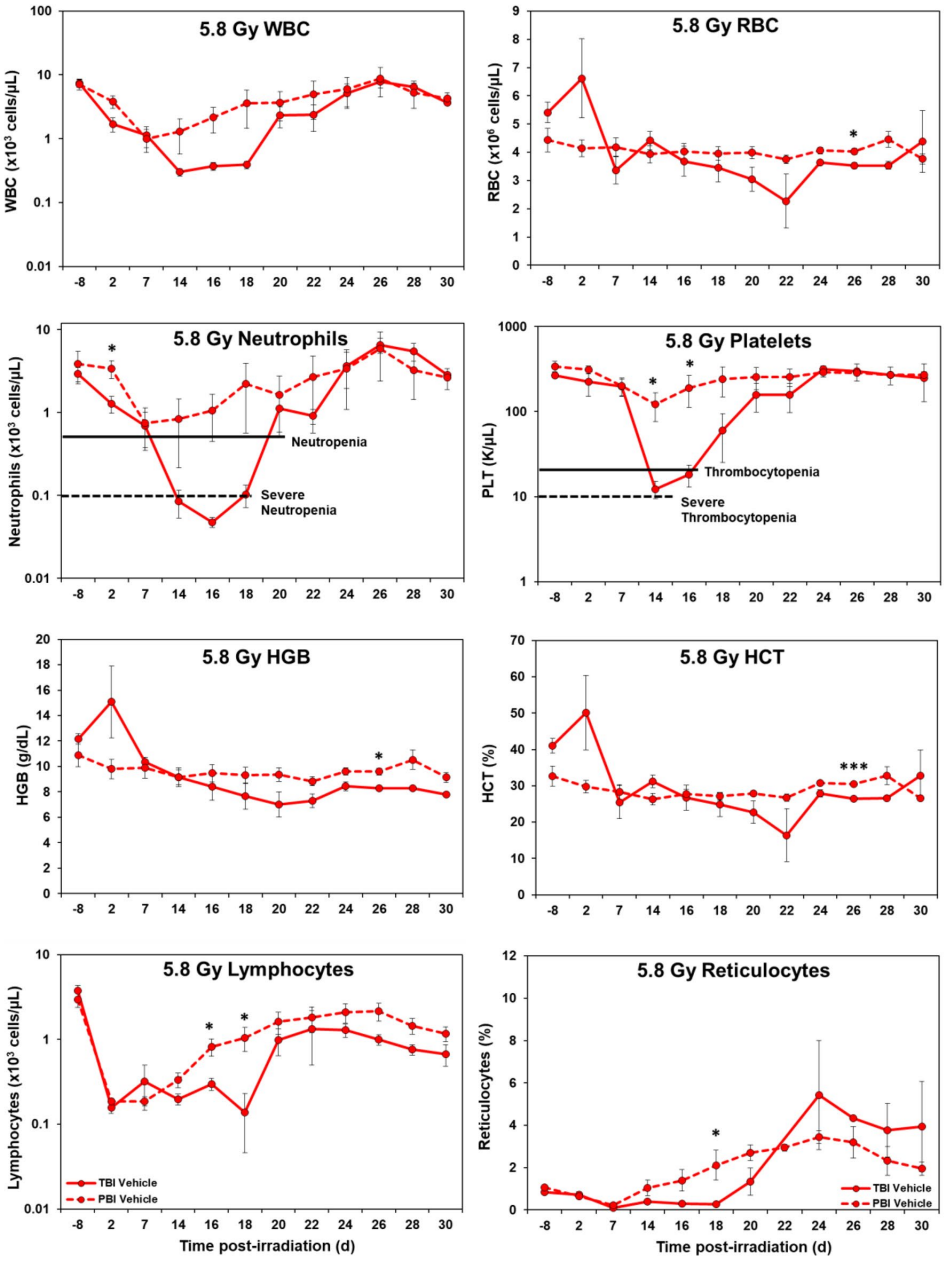
Effects of 5.8 Gy total-body and partial-body irradiation on vehicle-treated NHPs. The data for each time point is presented as the mean for each group. There was a total of 7 NHPs; 4 in the TBI group and 3 in the PBI group. Analysis of variance was performed with SPSS to detect significant differences between TBI and PBI groups. The number of asterisks corresponds to the level of statistical significance as follows: *p < 0.05, ***p < 0.001.

*Hematology.* Marked differences were noted in the temporal patterns of peripheral blood responses of the test animals under the two IR exposure/dose regimens, as well as those animals receiving either the control vehicle treatment or the test agent, GT3 (Figs. 1, 2, 3, and 4). In general, the extent and duration of acute cytopenia (e.g., leukopenia, neutropenia and thrombocytopenia) were exaggerated and prolonged not only by the higher radiation exposure level (5.8 Gy), but also by the lack of use of the prospective, injury-mitigating GT3. The time-dependent recovery from radiation exposure-induced cytopenia tended to exhibit reciprocal patterns relative to radiation dose, treatment applied, and the extent of bodily exposure (i.e., TBI vs. PBI). Of note were the less pronounced changes in other erythroid-related blood counts/parameters (RBCs, HGB, HCT, etc.) following lower, non-lethal exposures of 4 Gy as compared to those observed at the higher exposure levels of 5.8 Gy.

### Pathological assessments

*Gross pathological observations.* General pathological observations of all euthanized animals at the time of necropsy were recorded and listed in Table 5. In general, the ‘body-condition score’ (BCS) tended to be lower, albeit marginally lower, in those animals exposed to the higher dose of IR (5.8 Gy). Further, lower (from normal) BCS appeared related not only to the IR regimen applied (TBI vs. PBI), but also whether the animals were treated with test agent, GT3 or solely its vehicle (Table 5). Further, as per the BCS data, the type/frequency of gross organ/tissue pathology noted during necropsy seemed to not only be related to the IR protocol and IR dose applied, but also whether the animals were pretreated with GT3 or its vehicle (Table 5).

#### Organ system/tissue pathology

**Figure 5:**
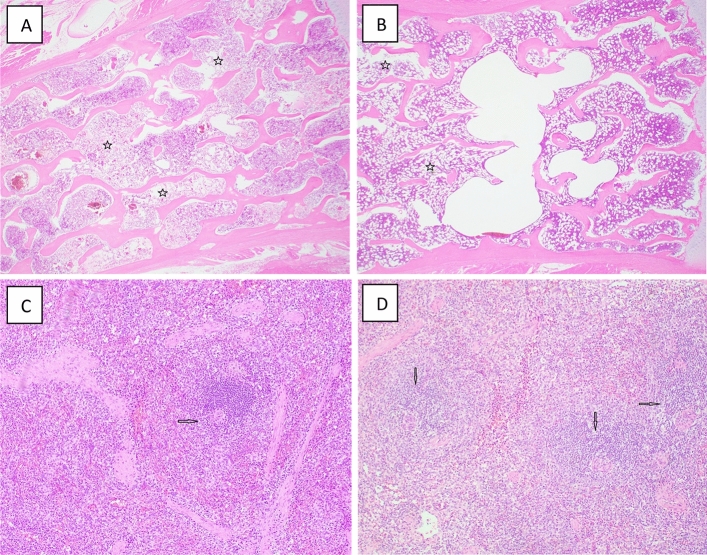
Representative photomicrographs of lymphohematopoietic organs collected from NHPs exposed to 4 or 5.8 Gy TBI. (**A**) 1607030F TBI 5.8 Gy, Sternum, 200x, regional areas of moderate cellular losses (stars). (**B**) 4.0 Gy TBI, Sternum, multiple areas of mild cellular losses (stars). (**C**) 1608016F 5.8 Gy TBI Veh, Spleen, 100x, rare white pulp and markedly depleted of cellular components arrow). (**D**) 1603138F 5.8 Gy TBI GT3, Spleen, 100x, multiple white pulps are moderately depleted of cellular components (arrows).

*Lymphohematopoietic system/sternum.* Direct, histopathologic observations of the sternum generally revealed a degree of hypoplasia within the marrow space of the sternum tissues of all the IR exposed animals. In general, hypoplasia was more evident and pronounced within tissues of animals exposed at the higher dose level of 5.8 Gy (relative to the lower dose level of 4 Gy). By comparison, the use of PBI (by contrast to TBI) appeared to lessen somewhat the severity of marrow hypoplasia, as did the test GT3 pretreatment (Fig. 5).

*Lymphohematopoietic system/spleen.* Histopathologic examination of splenic tissues of all the IR exposed animals showed varying degrees of cellular depletion of the white pulp, with greater losses noted in those animals exposed TBI at the higher doses (5.8 Gy vs. 4 Gy) (Table 6). In general, pretreatments with test medicinal, GT3, appeared to lessen somewhat this depletion- effect.

**Figure 6:**
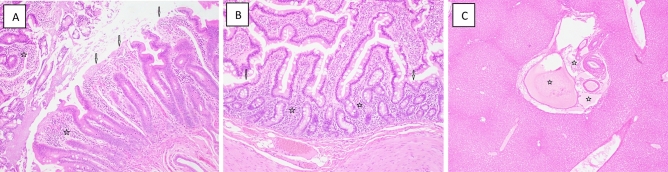
Representative photomicrographs of digestive organs collected from NHPs exposed to 4 Gy TBI or PBI. (**A**) 1606069 4.0 Gy TBI Veh, Duodenum, 100x, moderate loss and fusion of villi (arrows) and mild inflammatory cellular infiltrates (stars). (**B**) 1603168F 4.0 Gy PBI GT3, Duodenum 100x, mild loss and fusion of villi (arrows) and mild inflammatory cellular infiltrates (stars). (**C**) 1707119M 4.0 Gy PBI Veh, Liver 40x, multiple cystic dilation centered on portal area (stars).

*Gastrointestinal system/small intestine/duodenum.* The intestinal duodenal tissues of all animals in all groups showed varying degrees of tissue pathology, as evidenced by the noted loss of villi, villus fusion, decline in the villus to crypt cell ratio, as well as the presence of inflammatory cellular infiltrates (Fig. 6). In general, the later changes were marginally greater following TBI when compared to PBI and were somewhat lessened with GT3 pretreatments (Table 6).

*Gastrointestinal system/small intestine/jejunum.* The extent of pathologic changes noted within the jejunum was limited and not as obvious as those seen within the duodenum. Nevertheless, loss of villi, coupled with villus fusion and declining villi/crypt radios were indeed noted following both the high and the low levels of TBI and PBI exposures (4 Gy & 5.8 Gy). With GT3 pretreatments, the extent of these changes appeared somewhat less, but variable and unremarkable (Table 6).

*Gastrointestinal system/small intestine/ileum.* The histopathologic changes noted within the ileum were consistent with those changes noted within the duodenum and the jejunum. Substantial losses of villi, coupled with the extent of villus fusion and declining villi/crypt radios were noted following both the high and the low levels of TBI (Table 6). The latter dose-dependent changes were generally noted under PBI as well, with the exceptions being the marginal decline in villus fusion along with slight rise in the villus/crypt cell ratio following the higher exposure (5.8 Gy) (Table 6). With GT3 pretreatments (compared to vehicle alone pretreatments), the extent of these changes appeared somewhat less, but not markedly so (Table 6).

*Gastrointestinal system/large intestine/colon.* Clear and consistent differences in the histologic patterns of pathology within the sampled colonic tissues were not seen based on the loss or dilation of intestinal crypt cells, relative to the extent or IR exposure. Similarly, pretreatments with the test agent, GT3, had little noticeable effect on these colonic structures (Table 6).

*Digestive system/liver.* Pathologic lesions within liver were evident for the irradiated animals, but these lesions (e.g., cystic degeneration, inflammatory infiltrates) were generally unremarkable and lacked definite association with the IR regimen or the type of pretreatment (Fig. 6 and Table 6).

**Figure 7:**
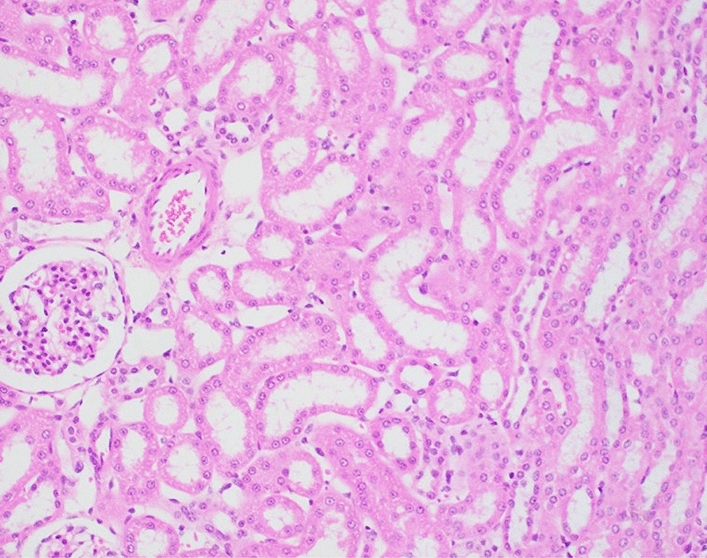
Representative photomicrograph of a kidney collected from an NHP exposed to 5.8 Gy TBI. 1608039M, 200x, area of tubular epithelial attenuation with moderately dilated lumen.

*Urinary system/kidney.* Regardless of the dose or regimen of radiation exposure, or the pretreatment applied, all kidney sections demonstrated some degree of tubular degeneration, characterized by variably dilated lumen lined with attenuated tubular epithelial cells. However, these test variables (i.e., dose and regiment of exposure, type of pretreatment) appeared to have only marginal impact on the changes noted, with the exception of the apparent lessening of tubular epithelial degeneration following GT3 pretreatments (Fig. 7 and Table 6).

*Respiratory system/pulmonary tissue/lung.* There were slight but discernable histopathologic differences in alveolar septal degeneration within lung sections of 4.0 and 5.8 Gy TBI vehicle-treated animals compared to the GT3-treated animals (Table 6). The degree of septal degeneration was marginally greater for the vehicle-treated groups compared to the GT3-treated NHPs. These differences appeared significant and might well have been associated with GT3 pretreatments.

**Figure 8:**
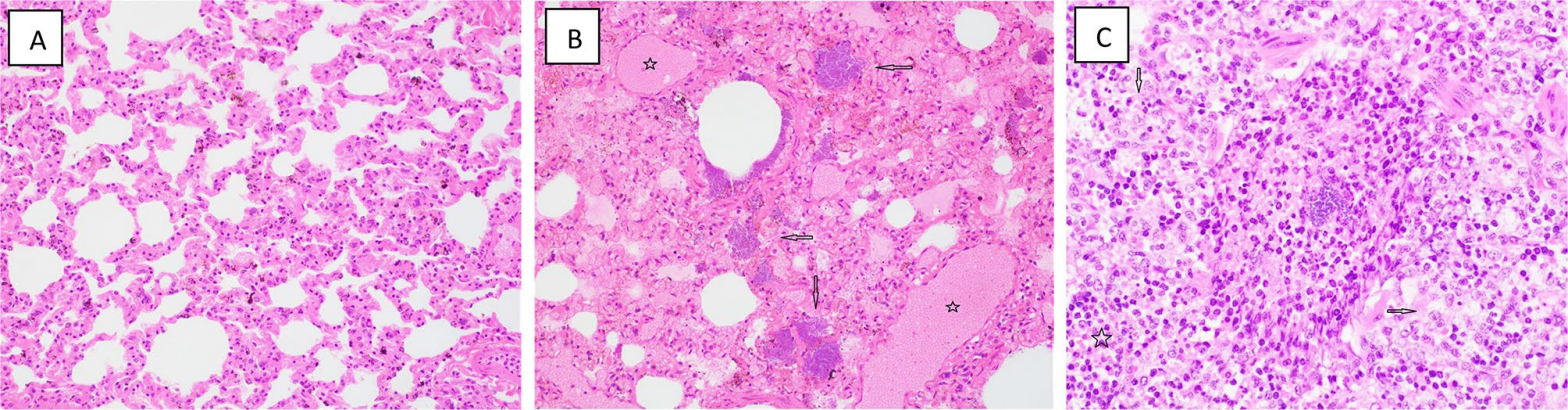
Representative photomicrographs of respiratory organs collected from NHPs. (**A**) RA3228F 4 Gy TBI, Lung, 200x, diffusely and moderately degenerated septum. (**B**) RA1251M Control, Lung, 400x, multiple large bacterial colonies (arrows) and medium vessels congested (stars). (**C**) 1607030F 5.8 Gy TBI, Lung, 400x, foci of lymphocytes (stars) and histiocytes (arrows).

Within select animals, most notably those within the 5.8 Gy TBI cohort, septal regions of the lung had moderately high cellular infiltrates comprised mainly of lymphocytes and histocytes within their tissues (Fig. 8). Of note, GT3-pretreated animals did not exhibit such cellular infiltrates. Further, two of the vehicle-pretreated animals exhibited locally extensive bronchointerstitial pneumonia, although in one of these animals there was minimal involvement of adjacent alveolar septal area. No septal cellular infiltration was observed in the PBI groups.

## Discussion

This study focused primarily on the histopathological nature of IR-associated injuries induced at two levels of IR-exposure, namely a relatively low, non-lethal level (4 Gy) and a higher, level (5.8 Gy) that would elicit ~ 30% lethality (within ~ 60 days) (Figs. 5, 6, 7, and 8). Two types of exposure regimens were also employed, namely a ‘total body-type’ exposure (TBI) and a ‘partial body-type’ exposure (PBI), with the later regimen designed to partially limit the exposure to ~ 95% of the full dose to the widely distributed blood-forming tissues of the body (or conversely, a sparing of ~ 5% of the marrow tissue from IR). Recently in a related reported ‘side-arm’ study, we demonstrated that the GT3 mediated hematopoietic recovery in these total- or partial-body exposed NHPs^42,43^. In this study, flow cytometry was used to immunophenotype the bone marrow (BM) lymphoid cell populations, while select, hematopoietic progenitor populations (HSCs, CFUs) were assessed periodically over a 30-day post-exposure period using standard assays. Significant changes in the frequencies of B and T-cell subsets were noted, along with changes in the self-renewing capacity of HSCs. Importantly, GT3 accelerated the recovery in CD34^+^ cells, increased HSC function as shown by improved recovery of CFU-granulocyte macrophages (CFU-GM) and burst-forming units erythroid (B-FUE), and aided the recovery of circulating neutrophils and platelets. In yet other related ‘side-arm’ analyses, we evaluated metabolomic and lipidomic profiles in multiple tissues (jejunum, lung, kidney, and spleen) of these tocotrienol-treated and irradiated animals^58^. Results indicated that IR-induced metabolic changes were partially mitigated by GT3-prophylaxis, with lung and splenic tissues being most responsive. Further, these analyses suggest that a prophylactic administration of GT3 might provide a simple solution in combating this type of metabolically-based radiation injury.

In general, and not surprisingly, the types and severity of injuries noted within the various organs/tissues examined varied with some exhibiting little change (e.g., liver) while others were more extensively altered (e.g., bone marrow). This is consistent with the well-recognized, well-documented radiobiological principle, namely that organ systems that are functionally dependent on high cell replacement rates tend to be more radiosensitive than those organ systems with lower rates. The lymphohematopoietic system represents the ‘archetype’ of a ‘high cell turnover’ organ system that functionally depends on amplifying small numbers of primitive, immature progenitors and their lineage-determined progeny through multiple rounds of reproduction, followed by differentiation and maturing of those amplified cells into fully functional blood cells (e.g., granulocytes, erythrocytes, etc.). It is these progenitor subpopulations that serve to not only to drive self-renewal of the tissue/organ system, but also is the primary determinant of its sensitivity to acute IR. The marked histopathology noted within the marrow sternum specimens of the irradiated animals clearly manifests this tissue’s relatively high radiosensitivity, as does the loss of white pulp within the splenic tissues. Further, these pathologies appear more severe following TBI (relative to the PBI regimen) at the higher of the two exposure levels applied (5.8 Gy vs. 4 Gy). Of particular note, the severity of these pathologies seemed to have lessened by the GT3 nutraceutical pretreatments. Results of the periodic clinical monitoring of these irradiated animals, especially in terms of the changing blood parameters, tend to support overall these pathologic trends.

In a similar fashion, the integrity and function of gastrointestinal system (GIS) is dependent on a comparable system of progenitor-dependent, self-renewing epithelia that lines outer mucosal walls of the intestine. Like the hematopoietic system, moderate doses of IR tend to disrupt the function of this cell renewing system, resulting in gastrointestinal injury that manifests both structurally and functionally, as exemplified by the histopathology of villus and crypt cell structures within the various segments of the small intestine. Again, like the lymphohematopoietic tissues, the GIS response to acute irradiation was modified not only by the IR dose, but also by the exposure regimen and by the applied nutraceutical pretreatment.

In contrast to the pathologic responses of these two robustly, self-renewing tissues (lymphohematopoietic and gastrointestinal tissues) and the modification of those responses relative to radiation dose, type of exposure regimen, or nutraceutical prophylaxes, the remaining tissues examined, namely liver, kidney and lung, with significantly lower levels of self-renewal, appeared less responsive in terms of the severity of the pathologies observed. This is not to say that these tissues were unresponsive to IR, for indeed they clearly harbored IR-associated lesions, but that these lesions appeared not to be acute or life-threatening in the short term. Through previous work^59–61^, it is well recognized that seemingly innocuous IR lesions within weakly, self-renewing tissues such as the kidney or the liver, can progress with time into pathologies that are more severe and more lethal. Such pathologies are often characterized or labeled as being ‘delayed or late-arising’ by nature and again, by contrast, can arise either stochastically (e.g., IR induced cancers) or deterministically (e.g., tissue fibrosis).

As mentioned earlier, part of the study attempted to examine the possible ‘pathology-sparing effect’ of the PBI-type IR exposures and it was found that not all of the tissues examined were spared (or affected) equally or in the same manner. For example, sternal bone marrow, splenic white pulp, duodenal intestine, and lung exhibited a lessening of pathology induced at the higher exposure level (5.8 Gy) under PBI as compared to the same dose delivered TBI. By contrast, the kidneys of animals subjected to PBI or TBI exhibited little difference in terms of the extent of tubular degeneration.

We used high level cobalt-60 facility for TBI. Cobalt facility has a panoramic field. Even if one tries to protect any part of the body, one cannot provide full protection. Thus, for PBI, LINAC is used because LINAC provides a collimated beam and the irradiation field is limited. Therefore, one can place in the field only part of the body one is interested in. Since same dose of radiation in total-body and partial-body with 5% bone marrow sparing will have different lethality, use of two different sources was not a major issue.

Finally, a major goal of the study was to evaluate the capacity of a nutraceutical, gamma tocotrienol or GT3, when used prophylactically to suppress or to limit IR-associated pathologies. Although the radioprotective effect of GT3 was somewhat less than we had expected, we did observe an apparent lessening of pathology within select tissues/organ systems when the GT3 was administered. The improved blood and marrow pictures of the GT3 treated animals, relative to the untreated control animals, tend to support our working hypothesis; namely that this agent, GT3, has radioprotective properties when used under defined exposure conditions. It should also be noted that the only two acutely irradiated animals in vehicle treated group became moribund prior to scheduled euthanasia date. This number likely would have been higher others not a sizable fraction of the animals not been pretreated with GT3. Admittedly, the number of animals here are too small to preclude a definitive conclusion on the agent’s capacity to reduce IR induced lethality: without doubt, additional studies are needed and are currently in the planning stage.

### Supplementary Information

Supplementary Tables.

## Data Availability

All relevant data are within the manuscript.
