# Supplementary material for: Pathology of acute sub-lethal or near-lethal irradiation of nonhuman primates prophylaxed with the nutraceutical, gamma tocotrienol
Source: Sci Rep. 2024 Jun 10;14:13315. doi: 10.1038/s41598-024-64102-8 (PMC11164941; doi:10.1038/s41598-024-64102-8)
Supplement: Supplementary file 1 — Supplementary Tables. [file 41598_2024_64102_MOESM1_ESM.pdf]

Supplementary Table 1. Body weights of NHPs subjected to 4 or 5.8 Gy TBI or PBI and treated with GT3 or vehicle

Radiation Dose	Exposure Level	Treatment	Euthanasia (d)	NHP#	Sex	Age (years)	Body weight		
							Initial (kg)	d 30	% Change
4 Gy	PBI	Vehicle	30	1603102	F	3.9	4.66	4.74	1.72
				1707119	M	3.8	5.42	5.36	-1.11
				1704305	M	2.8	4.32	4.36	0.93
				1603026	F	3.9	5.98	5.84	-2.34
		GT3		1603079	M	3.9	5.28	5.18	-1.89
				1704119	M	3.8	5.38	5.42	0.74
				1606088	F	3.6	5.92	5.86	-1.01
				1603168	F	3.9	5.06	5.62	11.07
	TBI	Vehicle	30	RA2695	M	4.7	9.44	9.04	-4.24
				1606069	M	3.6	5.02	4.98	-0.80
				RA3228	F	4.1	6.76	6.40	-5.33
				1608010	F	3.5	4.38	4.38	0.00
		GT3		RA3275	M	3.9	6.14	6.36	3.58
				1606093	M	3.6	4.74	5.02	5.91
				RA2770	F	5.1	4.90	4.88	-0.41
				1607012	F	3.5	5.86	5.44	-7.17
5.8 Gy	PBI	Vehicle	30	1608017	M	3.5	5.42	5.48	1.11
				1608018	F	3.5	5.16	5.00	-3.10
				1604188	F	3.8	5.52	5.68	2.90
		GT3		1608007	M	3.5	5.00	4.76	-4.80
				1708011	M	2.5	5.10	5.06	-0.78
				1603104	F	3.9	4.10	4.04	-1.46
				1608006	F	3.5	5.00	5.56	11.20
	TBI	Vehicle	30	1608039	M	3.5	4.94	4.88	-1.21
				1603015	M	3.9	4.72	4.44	-5.93
			22	1607030	F	3.5	3.84	3.90*	1.56
			21	1608016	F	3.5	5.38	4.55*	-15.43
		GT3	30	1703269	M	2.9	5.00	4.72	-5.60
				1607029	M	3.5	4.52	4.56	0.88
				1603138	F	3.9	4.62	4.50	-2.60
				1603030	F	3.9	4.78	4.76	-0.42

* These weights were recorded on d 20 due to early euthanasia.

d, day

Supplementary Table 2. Body temperatures of NHPs subjected to 4 or 5.8 Gy TBI or PBI and treated with GT3 or vehicle

Radiation Dose	Exposure Level	Treatment	Euthanasia (d)	NHP#	Sex	Age (years)	Temperature (Celsius) recorded post-irradiation (d)											
							-8	2	7	14	16	18	20	22	24	26	28	30
4 Gy	PBI	Vehicle	30	1603102	F	3.9	37.72	37.22	38.39	38.50	38.72	38.22	38.33	38.56	38.67	38.50	38.61	38.50
				1707119	M	3.8	39.06	38.83	39.33	39.28	39.22	39.78	39.56	39.72	39.56	40.28	38.72	40.39
				1704305	M	2.8	38.06	38.11	39.17	38.67	38.72	38.78	39.00	39.28	39.22	39.28	39.06	38.67
				1603026	F	3.9	38.67	38.28	38.39	38.06	39.33	39.17	39.28	39.61	39.50	39.56	39.44	38.61
		GT3		1603079	M	3.9	39.06	38.56	39.11	39.06	38.39	39.33	38.61	39.00	39.28	38.06	37.61	38.94
				1704119	M	3.8	38.78	39.22	39.50	39.50	39.83	40.06	39.83	39.89	40.33	40.44	39.50	40.17
				1606088	F	3.6	38.50	38.83	40.06	39.72	39.33	39.22	39.17	39.39	38.67	38.61	39.61	39.67
				1603168	F	3.9	38.83	38.89	39.28	39.22	40.00	39.78	39.72	39.94	39.28	39.17	39.72	40.39
	TBI	Vehicle	30	RA2695	M	4.7	38.22	39.11	38.44	38.83	37.44	37.39	38.94	38.50	37.39	38.28	38.72	39.28
				1606069	M	3.6	38.22	39.50	39.50	38.89	39.50	39.89	39.33	39.17	39.78	39.83	40.50	39.28
				RA3228	F	4.1	40.72	39.61	38.50	39.39	38.44	37.39	39.11	39.22	38.06	38.72	38.72	38.72
				1608010	F	3.5	38.67	38.28	37.72	38.28	38.50	38.44	39.06	38.89	38.67	38.67	38.67	38.00
		GT3		RA3275	M	3.9	39.17	39.28	38.50	38.83	38.11	37.50	38.72	38.50	38.39	38.83	38.06	39.11
				1606093	M	3.6	38.61	38.94	39.06	38.72	39.33	39.44	39.44	39.06	39.67	39.44	39.39	39.11
				RA2770	F	5.1	39.83	38.72	39.50	38.94	37.89	38.39	39.11	39.06	37.72	38.22	38.28	39.06
				1607012	F	3.5	38.94	38.56	38.72	38.89	39.44	39.22	39.44	40.11	39.44	39.56	39.89	39.39
5.8 Gy	PBI	Vehicle	30	1608017	M	3.5	38.72	36.56	36.89	36.94	39.11	39.33	39.11	39.17	39.50	40.72	40.78	38.83
				1608018	F	3.5	39.11	36.28	37.06	37.66	40.06	39.78	39.89	40.00	40.44	40.94	40.06	39.27
				1604188	F	3.8	38.50	38.39	37.11	37.72	39.28	39.17	38.78	39.17	40.44	40.56	39.28	38.20
		GT3		1608007	M	3.5	39.39	38.72	38.89	36.77	40.22	40.06	39.89	40.33	40.83	41.06	40.50	38.72
				1708011	M	2.5	38.89	38.11	38.72	38.27	38.33	39.17	39.11	39.28	39.39	40.00	39.83	37.88
				1603104	F	3.9	38.89	36.33	38.06	37.72	38.94	38.72	39.06	39.00	39.94	39.72	39.33	38.72
				1608006	F	3.5	38.94	37.61	38.39	37.77	39.28	39.44	39.17	39.33	39.50	39.83	40.83	39.50
	TBI	Vehicle	30	1608039	M	3.5	38.22	39.11	39.00	39.39	40.06	40.17	40.61	40.50	40.78	41.56	41.06	37.72
				1603015	M	3.9	37.56	38.50	38.83	38.39	39.33	39.22	39.39	40.06	39.94	39.89	38.78	38.39
			22	1607030	F	3.5	38.56	38.67	37.89	38.22	39.00	39.56	39.72	ND	ND	ND	ND	ND
			21	1608016	F	3.5	37.50	38.39	38.06	37.67	39.50	40.00	38.50	ND	ND	ND	ND	ND
		GT3	30	1703269	M	2.9	38.00	37.00	38.22	38.50	39.50	39.72	39.33	39.78	40.50	41.06	40.89	39.39
				1607029	M	3.5	38.17	37.56	38.50	37.72	39.72	40.50	39.83	40.33	40.67	40.83	40.22	38.00
				1603138	F	3.9	39.56	38.22	38.94	39.61	39.78	39.44	39.50	39.72	40.22	39.67	39.56	38.50
				1603030	F	3.9	39.67	38.39	38.89	37.72	38.89	38.28	38.83	39.33	39.11	38.56	39.33	38.94

d, day; ND, no data

Supplementary Table 3. Heart rates of NHPs subjected to 4 or 5.8 Gy TBI or PBI and treated with GT3 or vehicle

Radiation Dose	Exposure Level	Treatment	Euthanasia (d)	NHP#	Sex	Age (years)	Heart Rate (BPM) recorded post-irradiation (d)							
							-8	16	18	20	22	24	26	28
4 Gy	PBI	Vehicle	30	1603102	F	3.9	262	257	204	144	144	132	148	136
				1707119	M	3.8	256	220	260	232	224	236	250	212
				1704305	M	2.8	232	200	230	250	188	196	240	204
				1603026	F	3.9	244	208	250	260	200	188	215	208
		GT3		1603079	M	3.9	180	230	243	188	136	104	124	144
				1704119	M	3.8	228	196	250	240	200	188	280	224
				1606088	F	3.6	248	192	220	208	240	212	230	200
				1603168	F	3.9	244	208	200	220	208	200	260	192
	TBI	Vehicle	30	RA2695	M	4.7	ND	ND	ND	ND	ND	ND	ND	ND
				1606069	M	3.6	ND	111	193	119	188	136	194	154
				RA3228	F	4.1	ND	ND	ND	ND	ND	ND	ND	ND
				1608010	F	3.5	ND	248	193	203	245	237	244	235
		GT3		RA3275	M	3.9	ND	ND	ND	ND	ND	ND	ND	ND
				1606093	M	3.6	ND	271	227	175	208	218	202	203
				RA2770	F	5.1	ND	ND	ND	ND	ND	ND	ND	ND
				1607012	F	3.5	ND	237	244	234	250	251	245	218
5.8 Gy	PBI	Vehicle	30	1608017	M	3.5	240	200	224	224	200	236	200	196
				1608018	F	3.5	232	196	260	204	196	188	208	220
				1604188	F	3.8	263	212	230	232	196	228	208	212
		GT3		1608007	M	3.5	228	252	232	224	204	204	212	228
				1708011	M	2.5	208	224	230	200	220	208	196	216
				1603104	F	3.9	237	220	250	196	212	224	220	200
				1608006	F	3.5	224	204	228	192	200	188	240	196
	TBI	Vehicle	30	1608039	M	3.5	ND	204	200	214	130	204	234	284
				1603015	M	3.9	ND	206	188	192	194	189	153	141
			22	1607030	F	3.5	ND	276	240	238	ND	ND	ND	ND
			21	1608016	F	3.5	ND	251	300	253	ND	ND	ND	ND
		GT3	30	1703269	M	2.9	ND	249	206	154	181	170	188	186
				1607029	M	3.5	ND	216	244	109	241	218	229	184
				1603138	F	3.9	ND	207	213	204	161	219	192	176
				1603030	F	3.9	ND	211	273	218	233	250	228	177

d, day; ND, no data

Supplementary Table 4. Blood pressures of NHPs subjected to 4 or 5.8 Gy TBI or PBI and treated with GT3 or vehicle

Radiation Dose	Exposure Level	Treatment	Euthanasia (d)	NHP#	Sex	Age (years)	Blood pressure recorded post-irradiation (d)							
							-8	16	18	20	22	24	26	28
4 Gy	PBI	Vehicle	30	1603102	F	3.9	112/79	108/76	117/46	123/82	143/104	168/115	176/113	111/75
				1707119	M	3.8	130/58	140/99	153/97	143/93	119/99	124/95	11788	121/90
				1704305	M	2.8	165/88	127/92	157/91	134/100	145/73	157/119	136/70	132/88
				1603026	F	3.9	164/122	154/100	169/92	168/87	171/116	162/102	179/83	137/104
		GT3		1603079	M	3.9	136/94	99/42	106/76	120/72	89/52	125/91	146/82	138/91
				1704119	M	3.8	116/68	134/66	120/71	120/93	129/76	127/91	127/73	131/64
				1606088	F	3.6	163/119	145/78	144/65	133/85	125/85	155/55	142/84	133/82
				1603168	F	3.9	178/96	155/125	170/96	159/90	172/121	152/84	183/81	126/91
	TBI	Vehicle	30	RA2695	M	4.7	ND	ND	ND	ND	ND	ND	ND	ND
				1606069	M	3.6	ND	139/98	87/61	155/114	86/40	154/114	100/62	121/68
				RA3228	F	4.1	ND	ND	ND	ND	ND	ND	ND	ND
				1608010	F	3.5	ND	129/82	110/79	67/45	146/105	169/105	143/47	140/50
		GT3		RA3275	M	3.9	ND	ND	ND	ND	ND	ND	ND	ND
				1606093	M	3.6	ND	139/99	131/80	133/91	146/73	125/70	137/95	116/88
				RA2770	F	5.1	ND	ND	ND	ND	ND	ND	ND	ND
				1607012	F	3.5	ND	89/60	105/87	117/84	101/71	165/85	121/75	126/87
5.8 Gy	PBI	Vehicle	30	1608017	M	3.5	138/88	132/64	137/63	108/65	116/82	135/74	126/74	136/67
				1608018	F	3.5	138/78	149/91	175/122	149/68	127/99	112/77	120/71	126/68
				1604188	F	3.8	124/92	140/86	140/89	146/103	137/84	131/82	112/78	116/66
		GT3		1608007	M	3.5	126/66	131/69	123/87	140/105	137/87	100/72	144/94	169/90
				1708011	M	2.5	112/59	123/101	136/59	110/59	126/94	138/96	139/64	131/47
				1603104	F	3.9	151/74	129/69	151/84	146/110	116/85	143/86	105/61	103/67
				1608006	F	3.5	148/77	132/71	135/99	114/93	142/79	166/134	127/53	135/78
	TBI	Vehicle	30	1608039	M	3.5	ND	121/83	139/71	86/59	138/120	132/73	109/77	107/68
				1603015	M	3.9	ND	106/73	77/59	97/58	94/62	116/48	117/87	105/61
			22	1607030	F	3.5	ND	126/103	69/38	75/46	ND	ND	ND	ND
			21	1608016	F	3.5	ND	75/54	108/43	79/31	ND	ND	ND	ND
		GT3	30	1703269	M	2.9	ND	142/66	71/40	132/75	130/86	100/63	114/47	111/62
				1607029	M	3.5	ND	102/85	90/60	137/99	120/96	106/67	71/55	154/123
				1603138	F	3.9	ND	101/82	145/115	137/66	134/106	132/71	146/77	137/80
				1603030	F	3.9	ND	112/56	72/48	127/98	123/95	142/106	86/68	98/56

d, day; ND, no data
